# First Full Draft Genome Sequence of *Plasmodium brasilianum*

**DOI:** 10.1128/genomeA.01566-16

**Published:** 2017-02-09

**Authors:** Eldin Talundzic, Shashidhar Ravishankar, Vishal Nayak, Dhruviben S. Patel, Christian Olsen, Mili Sheth, Dhwani Batra, Vladimir Loparev, Fredrik O. Vannberg, Venkatachalam Udhayakumar, John W. Barnwell

**Affiliations:** a Malaria Branch, Division of Parasitic Diseases and Malaria, Center for Global Health, Centers for Disease Control and Prevention, Atlanta, Georgia, USA; b Atlanta Research and Education Foundation, VAMC, Atlanta, Georgia, USA; c School of Biology, Georgia Institute of Technology, Atlanta, Georgia, USA; d SRA International, Atlanta, Georgia, USA; e Torq Informatics, Mebane, North Carolina, USA; f Biotechnology Core Facility Branch, Division of Scientific Resources, Centers for Disease Control and Prevention, Atlanta, Georgia, USA

## Abstract

*Plasmodium malariae* is a protozoan parasite that can cause human malaria. The simian parasite *Plasmodium brasilianum* infects New World monkeys from Latin America and is morphologically indistinguishable from *P. malariae*. Here, we report the first full draft genome sequence for *P. brasilianum*.

## GENOME ANNOUNCEMENT

Human malaria can be caused by five parasite species called *Plasmodium falciparum*, *Plasmodium vivax*, *Plasmodium knowlesi*, *Plasmodium ovale*, and *Plasmodium malariae*. Reference genome sequences are now published for the first three species (1–3), and draft genome sequences for the latter three species have been made available online at http://biorxiv.org/content/early/2016/05/12/052696). *P. brasilianum* greatly resembles the quartan *P. malariae* morphologically, but early experimental cross-species infections were unsuccessful and thus were classified as distinct species (4). Recent work from the Venezuelan Amazon, however, has suggested that *P. brasilianum* is likely able to infect humans (5). Here, we report the first draft genome sequence for *P. brasilianum* based on Illumina short-read sequencing technology.

Genomic DNA was extracted from *ex vivo* mature schizont stage parasites of the Bolivian I strain of *P. brasilianum* using the Qiagen DNA blood kit (catalog no. 51104; Qiagen, CA, USA). Four separate genomic DNA libraries were prepared using the NEBNext Ultra library prep kit (catalog no. E7370S; New England BioLabs, MA, USA). Two of the libraries were sequenced on two separate runs on the MiSeq using the MiSeq 500-cycle reagent kit (catalog no. MS-102-2003; Illumina, CA). The remaining two libraries were sequenced on two separate runs on the HiSeq 2500 using the HiSeq 150-cycle reagent kit (FC-410-1002). For initial data quality filtering, BBduk (https://sourceforge.net/projects/bbmap/) was used to trim 5′ and 3′ ends (i.e., quality < 35), remove PhiX, sequencing indices, and adaptors, and discard reads less than 50 bp. Post-quality trim, potential host DNA contamination was removed using the *Saimiri* monkey reference genome (GenBank accession no. 1GCA_000235385.1). A total of 420,061,864 paired-end reads remained with a Q20 (i.e., base call accuracy) of 99.9% or greater from all four sequencing runs. Nuclear genome assembly of combined filtered reads was performed using CLC Genomics Workbench version 9.0. Quality assessment of the full genome assembly was done using QUAST (6). Gene prediction was performed using GeneMark-ES (7). The mitochondrial and apicoplast genomes were assembled using the Geneious *de novo* assembler using the circularize contigs with matching regions option (8). The *P. malariae* reference mitochondrial (GenBank accession no. AB354570) and apicoplast (GenBank accession no. AB649418) genomes were used to annotate all the genes for the respective *P. brasilianum* genomes. Both the mitochondrial and apicoplast genomes were 99.70% identical to the published *P. malariae* genomes.

The final assembly of the nuclear genome consisted of 963 contigs (mean coverage, 70×) comprising 31,73,3118 bp, with an *N*_50_ of 49,088 bp and G+C content of 25%. The largest contig was 344,535 bp. A total of 6,050 protein-encoding genes were predicted using GeneMark-ES. The complete *P. brasilianum* mitochondrial and apicoplast genomes are also included in this report.

### Accession number(s).

This whole-genome shotgun project has been deposited at DDBJ/ENA/GenBank under the accession no. MKLA00000000. The version described in this paper is version MKLA01000000.

## References

[1] Gardner MJ, Hall N, Fung E, White O, Berriman M, Hyman RW, Carlton JM, Pain A, Nelson KE, Bowman S, Paulsen IT, James K, Eisen JA, Rutherford K, Salzberg SL, Craig A, Kyes S, Chan MS, Nene V, Shallom SJ, Suh B, Peterson J, Angiuoli S, Pertea M, Allen J, Selengut J, Haft D, Mather MW, Vaidya AB, Martin DMA, Fairlamb AH, Fraunholz MJ, Roos DS, Ralph SA, McFadden GI, Cummings LM, Subramanian GM, Mungall C, Venter JC, Carucci DJ, Hoffman SL, Newbold C, Davis RW, Fraser CM, Barrell B 2002 Genome sequence of the human malaria parasite *Plasmodium falciparum*. Nature 419:498–511. doi:10.1038/nature01097. 12368864 PMC3836256

[2] Carlton JM, Adams JH, Silva JC, Bidwell SL, Lorenzi H, Caler E, Crabtree J, Angiuoli SV, Merino EF, Amedeo P, Cheng Q, Coulson RMR, Crabb BS, Del Portillo HA, Essien K, Feldblyum TV, Fernandez-Becerra C, Gilson PR, Gueye AH, Guo X, Kang'a S, Kooij TWA, Korsinczky M, Meyer EV-S, Nene V, Paulsen I, White O, Ralph SA, Ren Q, Sargeant TJ, Salzberg SL, Stoeckert CJ, Sullivan SA, Yamamoto MM, Hoffman SL, Wortman JR, Gardner MJ, Galinski MR, Barnwell JW, Fraser-Liggett CM 2008 Comparative genomics of the neglected human malaria parasite *Plasmodium vivax*. Nature 455:757–763. doi:10.1038/nature07327. 18843361 PMC2651158

[3] Pain A, Böhme U, Berry AE, Mungall K, Finn RD, Jackson AP, Mourier T, Mistry J, Pasini EM, Aslett MA, Balasubrammaniam S, Borgwardt K, Brooks K, Carret C, Carver TJ, Cherevach I, Chillingworth T, Clark TG, Galinski MR, Hall N, Harper D, Harris D, Hauser H, Ivens A, Janssen CS, Keane T, Larke N, Lapp S, Marti M, Moule S, Meyer IM, Ormond D, Peters N, Sanders M, Sanders S, Sargeant TJ, Simmonds M, Smith F, Squares R, Thurston S, Tivey AR, Walker D, White B, Zuiderwijk E, Churcher C, Quail MA, Cowman AF, Turner CMR, Rajandream MA, Kocken CHM, Thomas AW, Newbold CI, Barrell BG, Berriman M 2008 The genome of the simian and human malaria parasite *Plasmodium knowlesi*. Nature 455:799–803. doi:10.1038/nature07306. 18843368 PMC2656934

[4] Collins WE 1974 Primate malarias. Adv Vet Sci Comp Med 18:1–23. 4153614

[5] Lalremruata A, Magris M, Vivas-Martínez S, Koehler M, Esen M, Kempaiah P, Jeyaraj S, Perkins DJ, Mordmüller B, Metzger WG 2015 Natural infection of *Plasmodium brasilianum* in humans: man and monkey share quartan malaria parasites in the Venezuelan Amazon. EBioMedicine 2:1186–1192. doi:10.1016/j.ebiom.2015.07.033. 26501116 PMC4588399

[6] Gurevich A, Saveliev V, Vyahhi N, Tesler G 2013 QUAST: quality assessment tool for genome assemblies. Bioinformatics 29:1072–1075. doi:10.1093/bioinformatics/btt086. 23422339 PMC3624806

[7] Borodovsky M, Lomsadze A 2011 Eukaryotic gene prediction using GeneMark.hmm-E and GeneMark-ES. Curr Protoc Bioinformatics Chapter 4:Unit 4.6.1–4.6.10. 10.1002/0471250953.bi0406s35 PMC3204378 21901742

[8] Kearse M, Moir R, Wilson A, Stones-Havas S, Cheung M, Sturrock S, Buxton S, Cooper A, Markowitz S, Duran C, Thierer T, Ashton B, Meintjes P, Drummond A 2012 Geneious Basic: an integrated and extendable desktop software platform for the organization and analysis of sequence data. Bioinformatics 28:1647–1649. doi:10.1093/bioinformatics/bts199. 22543367 PMC3371832

